# Engineering of balanites aegyptiaca-derived SrO@biochar and SrO@biomass for nitrophenol removal from wastewater

**DOI:** 10.1038/s41598-025-26771-x

**Published:** 2025-12-15

**Authors:** Manal Fawzy, Rania Alzain, Abdelazeem S. Eltaweil, Eman M. Abd El-Monaem

**Affiliations:** 1 Environmental Sciences Department, Faculty of Science, Alexandria University, Alexandria, 21511 Egypt; 2 Green Technology Group, Faculty of Science, Alexandria University, Alexandria, 21511 Egypt; 3 Department of Engineering, Faculty of Technology and Engineering, University of Technology Applied Sciences, Ibra, Sultanate of Oman; 4 Department of Chemistry, Faculty of Science, Alexandria University, Alexandria, 21321 Egypt; 5 Advanced Technology Innovation, Borg El-Arab, Alexandria, Egypt

**Keywords:** Strontium oxide (SrO), Adsorption, Mechanistic study, O-nitrophenol, plant-derived nanoparticles, Isotherms, Cycling test, Biotechnology, Environmental sciences, Chemistry

## Abstract

This study reports a sustainable route for the synthesis of an eco-friendly adsorbent derived from *Balanites aegyptiaca* (Laloub) decorated with strontium oxide nanoparticles (SrO NPs) for the removal of ortho-nitrophenol (o-NP) from water. The plant extract was used as a reducing agent for the green synthesis of SrO-NPs, while the plant branches were used to prepare biomass (BM) and biochar (BC). The obtained composites (SrO-BM and SrO-BC) were characterized by XRD, FTIR, and SEM, that revealed that SrO NPs crystallinity, and confirmed the presence of functional oxygenated groups, with a uniform distribution of SrO-NPs on the BM and BC surfaces. A comparative study has been executed to evaluate whether converting BM into BC enhances its adsorption performance or represents wasted energy. The experimental results reflected that SrO-BC exhibited higher adsorption capacity of 335.57 mg/g toward o-NP compared with 234.74 mg/g for SrO-BM at optimum conditions of pH 5, 25 °C, and 0.5 g/L dosage. Adsorption isotherm models showed that the adsorption onto SrO-BM followed the Langmuir model, while that for SrO-BC followed Freundlich model with a pseudo-second-order behavior for both. Mechanistic insights suggested electron donor–acceptor interactions, hydrogen bonding, π–π stacking, and coordination bonding as governing pathways. Reusability tests confirmed adsorbent stability since they retained more than 80% of their removal efficiency after five cycles. These findings provide a green and eco-friendly route to fabricate efficient and reusable adsorbents derived from *Balanites aegyptiaca* with a superior adsorption potential of SrO-BC for sustainable wastewater treatment.

**Supplementary Information:**

The online version contains supplementary material available at 10.1038/s41598-025-26771-x.

## Introduction

Organic contaminants are one of the most dangerous sources of water pollution as they pose a threat to aquatic life as well as human health^1^. Nitrophenols represent one category of these organic contaminants due to their widespread applications, high toxicity, persistence, and the affinity to bioaccumulate^2,3^. Mouth irritation, blurred vision, kidney and liver damage, and systemic poisoning are some of the most common effects of the accumulated concentrations of nitrophenols^4^. It is well known that the nitro-group enhances the persistency of nitrophenols in water and soil^5^. Numerous technologies have been utilized to eliminate the impacts of nitrophenol pollutants, such as adsorption, Fenton oxidation, biological digestion, membrane filtration, and catalysis^6,7^. Among all, adsorption could be considered the most cost-effective and highly efficient technique, make it much favourable for water treatment from nitrophenols^8^.

The worldwide achievements in the application of traditional nanotechnology in many different scientific fields have happened at the cost of the environment^9,10^; traditional synthetic methods are known to carry environmental risks and are harmful to the ecosystem. These approaches involve the use of highly hazardous conditions, such as high temperatures, large amounts of organic solvents, and toxic reducing agents^11,12^. Nevertheless, the plant-based synthetic approaches to creating nanoparticles, known as green nanotechnology, have garnered significant attention from researchers. This is due to the global shift towards a more sustainable future, intending to reduce the negative impact of traditional synthesis methods. Moreover, plant extract acts as both a reducing and capping agent at the same time, which this dual benefit makes it a suitable precursor material for the fabrication of nanoparticles^13^. Recently, many studies have reported the removal of nitrophenol adsorption. For instance, Xu et al. (2025) focuses on sustainable fabrication of porous carbon derived from waste rubber. The fabricated porous carbon demonstrated excellent stability and reusability during many successive regeneration cycles^14^. In another study, Zhang, et al. fabricated magnetic ion exchange resins, focusing on the role of nitrophenol functional groups in the adsorption process^15^. One drawback is that the findings may not be directly applicable to all types of magnetic ion exchange resins, as the adsorption mechanism and efficiency can vary depending on the resin’s composition and structure. Gautam et al. (2025) investigated the potential of biochar derived from pineapple crowns and sweet lime fibres. A key limitation is the need to thoroughly investigate the long-term stability and reusability of these BCs in complex wastewater matrices^16^. Another research used pine bark cross linked with cyclodextrin (PB-CD) offers a promising solution However, the adsorption capacity is highly pH sensitive^17^.

Strontium oxide nanoparticles (SrO NPs) are deemed one of the promising transition metal oxide nanoparticles that have been inquired into manufacturing professions, for instance, super capacitors, gas sensor electrodes, solar cells, lithium-ion batteries, catalysts, and semiconductors^18,19^. In addition, SrO exhibited remarkable results in wastewater remediation since it is a self-supported cationic species, enabling it to uptake the anionic pollutants even with low concentrations^20^. Interestingly, SrO possesses a high tendency to integrate to boost its adsorption property, surface area, and recyclability. The scarcity of literature on the synthesis of SrO NPs and the limitations of current methods have prompted environmental scientists to explore utilizing plant extracts in the fabrication process^21^. Green synthesis procedures and moderate laboratory conditions for preparing SrO NPs could significantly reduce the environmental drawbacks of using un-eco-friendly methods^22,23^.

Biochar (BC) is a carbon-rich material manufactured by biomass pyrolysis under limited oxygen conditions^24^. Plentiful biomass sources are considered the primary substances for synthesizing BC, comprising sewage sludge, poultry manure, agriculture wastes, forestry residues, crops, and algae^25^. Thanks to the favourable properties of BC that granted it significant fame as an excellent adsorbent, including good mechanical strength, cost-effectiveness, porous structure, super-large surface area, and high adsorption capacity^26^. Notably, the oxygen functional containing BC enables it to functionalize or integrate to foster its adsorption performance^27,28^.

Balanites aegyptiaca (L.), commonly referred to as Laloub or Hegleig, is an indigenous tree species to Sudan. It belongs to the Zygophyllaceae family. This multipurpose tree has a significant cultural value and is widely utilized in traditional medicine. Balanites aegyptiaca also located within the tropical dry belt of North Africa, as well as in the arid regions of India and South Asia. The extract of Balanites aegyptiaca is characterized by a wide variety of phytochemicals, such as saponins, tannins, flavonoids, and cardiac glycosides. The occurrence of phenolic compounds, including tannins and flavonoids, is primarily linked to the application of this extract as a reducing agent^29^. These compounds function as reducing agents by donating electrons, which aids in the reduction of metal ions to their elemental state, subsequently resulting in the formation of nanoparticles.

The present research highlighted fabricating a new green adsorbent for removing o-nitrophenol (o-NP) from wastewater. *Balanites aegyptiaca* biomass (BM) was used for the first time as a reducing agent in the fabrication of SrO. In addition, the *Balanites aegyptiaca* residue was utilized to prepare BC. Although SrO has revealed remarkable adsorption performance in adsorbing diverse pollutants, it has not yet been used to remove o-NP. A complete comparison study proceeded between SrO-BC and SrO-BM composites toward the adsorption of o-NP molecules. The physical and chemical specifications of SrO-BC and SrO-BM composites were analysed using various characterization instruments, such as XPS, SEM, XRD, zeta potential, and FTIR. The adsorption experiments of o-NP were performed on SrO-BC and SrO-BM composites in a batch mode to record the best adsorption conditions of the o-NP molecules. A mechanistic study of o-NP onto SrO-BC and SrO-BM composites was proposed based on the kinetic and isotherm assessments and XPS analyses. A recycling experiment was done on SrO-BC and SrO-BM composites for five o-NP adsorption runs to ensure their durability.

## Experimental part

### Preparation of plant extract

Fresh specimens of *Balanites aegyptiaca* (L.), locally known as Laloub or Hegleig, were collected from Heglig district in South-West Sudan. This tree species is widely distributed in arid and semi-arid regions of Sudan (Al-Juhani et al.). Dr. Itmad Elhassan of Industrial Research and Consultancy Centre, Khartoum, Sudan collected the investigated plant specimens, from its natural habitats. A permission from Ministry of Environment, Forestry and Water was obtained and collecting processes followed guidelines of the International Union for Conservation of Nature (IUCN). Sample specimen of *Balanites aegyptiaca* was prepared and deposited in the herbarium of Khartoum University, Khartoum, Sudan with accession number K004508571.

*Balanites aegyptiaca* leaves are well rinsed with tap water, followed by soaking in distilled water, then dried in an oven at 50 °C until constant weight. The extract of *Balanites aegyptiaca* leaves was prepared as detailed in a previous study^30^. 5 g of plant leaves were added to 100 ml of distilled water and stirred for an hour at 70 °C. The obtained extract was filtered through filter paper to remove the excess plant leaves. The resulting aqueous extract was used as a reducing agent for the green synthesis of SrO nanoparticles.

### Fabrication of SrO NPs

The pre-prepared *Balanites aegyptiaca* extract was dropwise added to 100 mL of an aqueous solution of 0.1 M of SrCl_2_.6H_2_O while continuously stirring at 60 °C for 3 h. The pH of the Sr/plant extract solution was adjusted at pH = 10 using a few drops of concentrated NaOH. Next, the Sr(OH)_2_ particles were separated by centrifugation, washed with water and C_2_H_5_OH, and dried for 6 h at 90 °C. Finally, the dried Sr(OH)_2_ was calcinated at 700 °C for 2 h, forming a white powder of SrO NPs^31^.

### Preparation of biochar (BC)

Biochar was prepared through pyrolysis of the fine-grinded biomass of *Balanites aegyptiaca* branches for 45 min in a muffle furnace at 400 °C under limited oxygen conditions. The dark black powder of the *Balanites aegyptiaca-derived BC* was then stored in a falcon tube for further analysis and application^28^.

### Fabrication of SrO-BC and SrO-BM composites

The SrO-BC composite was created using a physical post-synthesis procedure. First, 0.1 g of SrO NPs were mixed with BC (10, 20, 25 wt%) in 20 mL of distilled water. The SrO/BC mixture was then sonicated for 30 min at room temperature. Afterward, the SrO/BC composite was collected and dried for 10 h at 50 °C in an oven^32^ .

Similarly, the SrO-BM composite was prepared using the same procedure as the SrO-BC composite but using *Balanites aegyptiaca* BM instead of BC.

### Batch adsorption process

The most appropriate conditions for adsorbing o-NP on the surfaces of the SrO-BC and SrO-BM composites were determined in a batch adsorption mode^33^. To determine the optimum pH, dose, temperature, and initial concentration, sequences of adsorption operations were performed at a range of pH varying from 3 to 11, dose range extended from 0.25 to 1.0 g/L, temperature range from 25 to 55 °C and initial concentration from 50 to 200 mg/L. During studying the ionic strength influence, different concentrations of NaCl were added to the adsorption media at the range of 0.2–1.0 mol/L. The residual o-NP concentrations were measured by a spectrophotometer at 344 nm. Afterward, the following equations were used to quantify removal percentage (R%) and adsorption capacity (q_t_).

1 $$\:{\text{q}}_{\text{t}}=\frac{{(\text{C}}_{\text{o}}-{\text{C}}_{\text{f}})\times\:\text{V}}{\text{m}}$$

2 $$\:\text{R}\:\text{\%}=\frac{{\text{C}}_{\text{o}\:}-{\text{C}}_{\text{f}}}{{\text{C}}_{\text{o}}}\:\times\:100$$

Where C_0_ and C_t_ are the concentration of the adsorbate at time (0) and time (t), respectively. V is the volume of adsorbate and m is the mass of adsorbent.

### Re-usability experiment

The recyclability of the Sr-BC and Sr-BM composites was tested by conducting five recycling runs. After the initial adsorption run, the composites were separated and soaked in 20 mL of concentrated sodium hydroxide with stirring for 30 min to remove the o-NP molecules attracted to their exterior surface. The Sr-BC and Sr-BM composite were washed with distilled water and dried for using them in the next cycle.

## Result and discussion

### Studying the characteristics of SrO-BC and SrO-BM

**Fig. 1:**
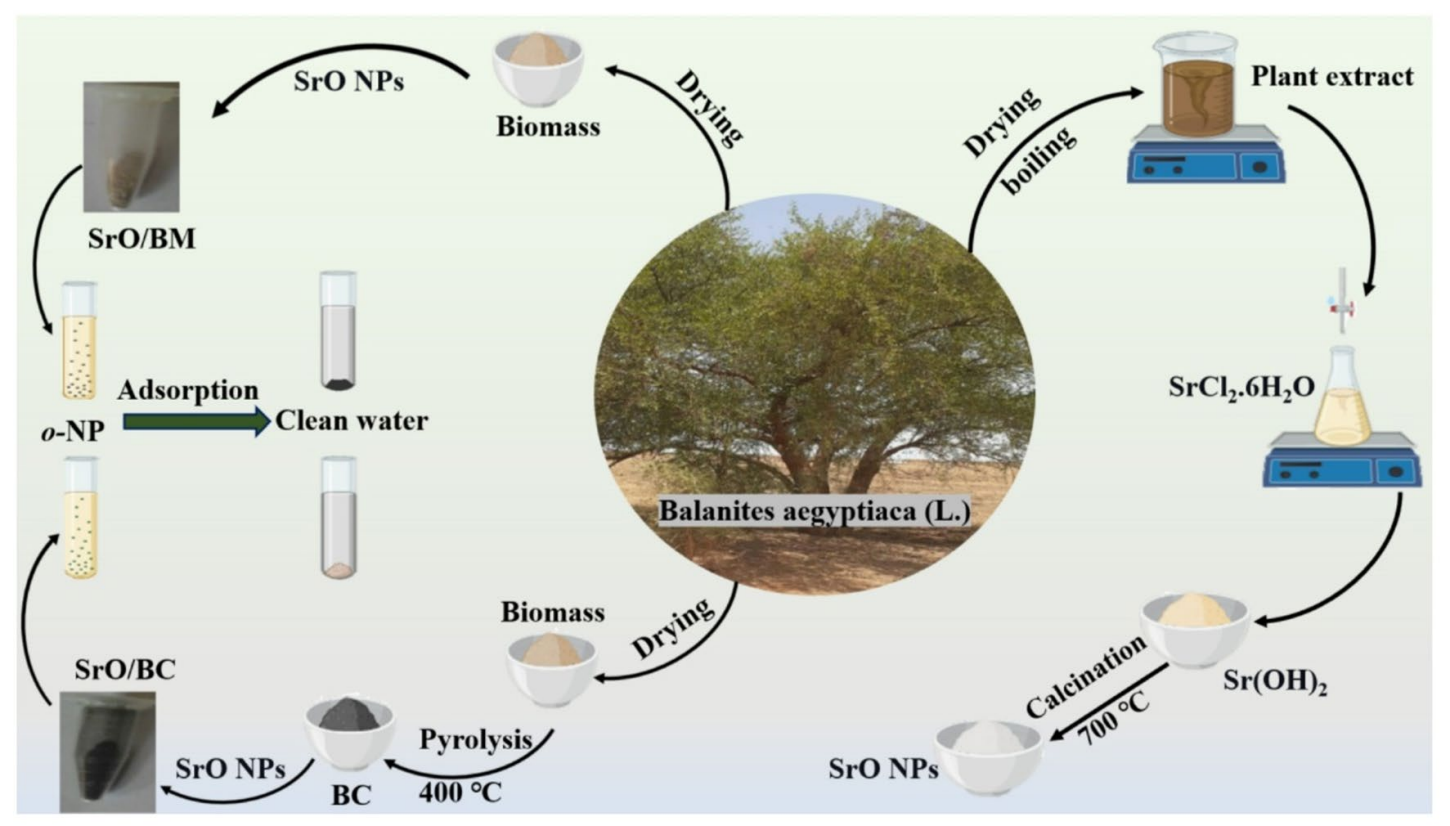
Eco-friendly route to fabricate Sr-BM and Sr-BC composites.

SrO-BM and SrO-BC composites were developed through a fully green and sustainable synthesis process, utilising *Balanites aegyptiaca* extract and biomass (Fig. 1). This eco-friendly approach highlights the potential of natural resources in advanced material fabrication. The plant extract served as a natural precipitant and stabilising agent for the synthesis of SrO NPs, eliminating the need for hazardous chemicals and aligning with the principles of green chemistry. To create the composites, part of the dried biomass was directly combined with SrO NPs to form SrO-BM, preserving the natural structure and properties of the biomass. In contrast, another portion of the biomass was pyrolyzed to produce biochar, pyrolysis transforms biomass into a more reactive and structured material (biochar), which then acts as a support or component in the co-precipitation process to form a composite with enhanced adsorption properties. enhancing its surface area and porosity, which was then mixed with SrO NPs to produce SrO-BC. This dual strategy not only valorises agricultural waste but also offers flexibility in tailoring the composites for diverse applications. The method demonstrates a sustainable and innovative pathway for producing functional materials with potential environmental and catalytic applications.

#### SEM

**Fig. 2:**
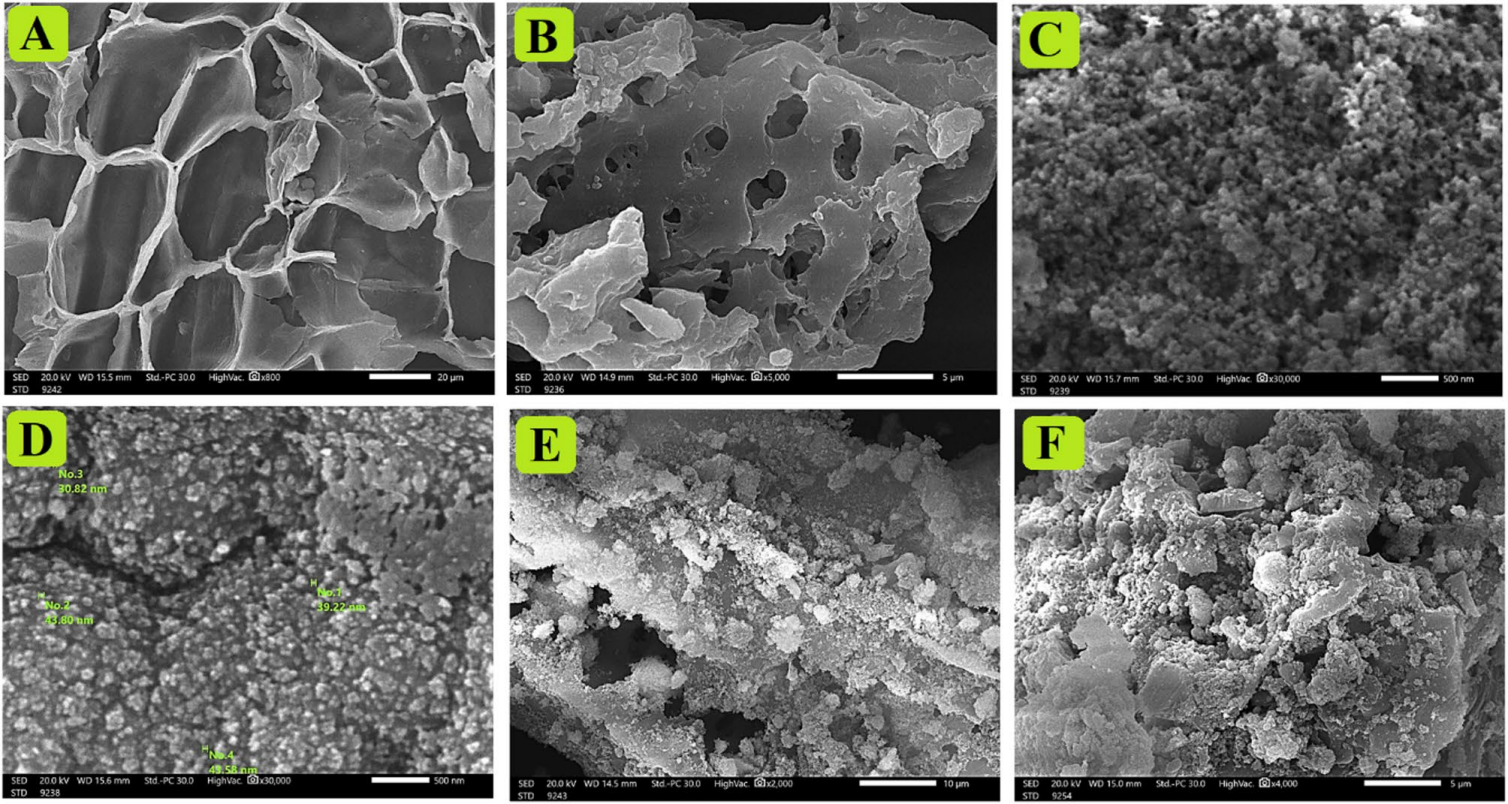
SEM of (**A**) BM, (**B**) BC, (**C**, **D**) SrO, (**E**) SrO-BC, and (**F**) SrO-BM.

The morphological shapes of BM, BC, SrO, SrO-BM, and SrO-BC are analyzed using SEM. The SEM of the authentic *Balanites aegyptiaca* BM elucidated interconnected loose cavities that look like a beehive (Fig. 2A). SEM of *Balanites aegyptiaca*-derived BC showed piled sheets with loose holes, as elucidated in Fig. 2B. The outer morphologies of *Balanites aegyptiaca* BM and its derived BC suggested their feasibility to work as excellent supporters. The outer-morphological shape of SrO NPs is like non-uniform spherical particles in a nanoscale size ranging from 30.82 to 43.80 nm (Fig. 2C, D). The SEM images of SrO-BC and SrO-BM observed the distribution of SrO nanoparticles over the surfaces of the composites, filling their pores, as demonstrated in Fig. 2E, F.

#### XRD analysis

**Fig. 3:**
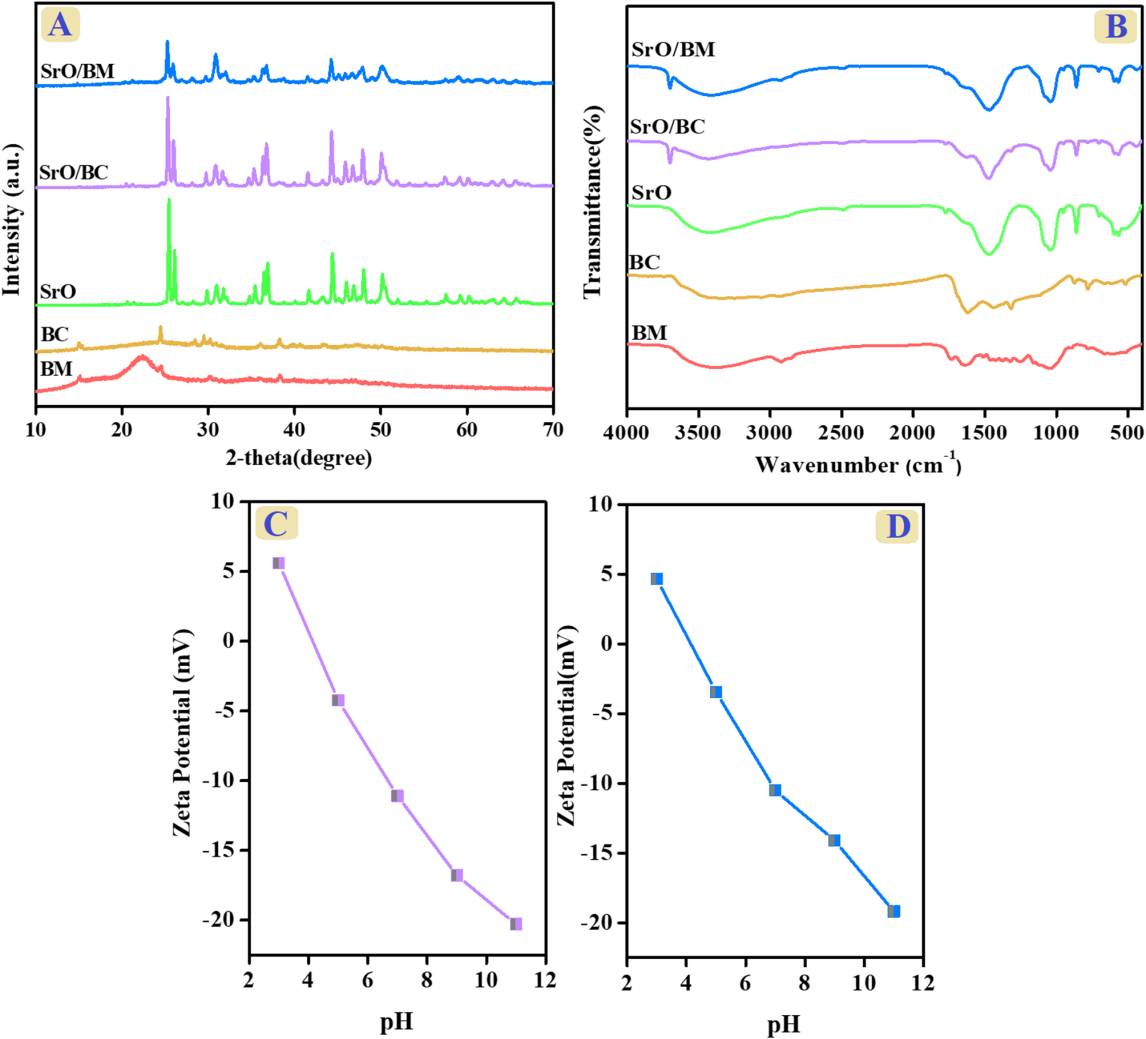
(**A**) XRD, (**B**) FTIR of BM, BC, SrO, SrO-BC, and SrO-BM, (**C**, **D**) ZP of SrO-BC, and SrO-BM.

The XRD diffractometer revealed the crystal structure of BM, BC, SrO, SrO-BC, and SrO-BM, as represented in Fig. 3A. The diffractogram of *Balanites aegyptiaca* BM depicted peaks at 14.8°, 21.1°, and 38.6° corresponding to crystal plans of (101), (002) and (111) are attributed to cellulose I, cellulose II, and hemicellulose, respectively. The diffractogram of *Balanites aegyptiaca*-derived BC clarified almost the same distinguishing XRD peaks of the BM, but with a noticeable enhancement in the crystallinity because of boosting the content of the inorganic residue after the combustion process. This observation aligns with the study by Sukarni et al., which confirmed the impact of combustion on reducing the crystallinity of biomass^34^. For SrO NPs, the XRD diffractogram elucidated peaks located at 2θ positions = 25.43º, 26.09º, 29.85º, 31.77º, 35.40º, 36.44º, 40.05º, 41.63º, 44.38º, 45.09º, 47.51º, 47.99º, 50.17º, 59.19º, 60.2º, and 64.29º accompanied with the crystal planes of (202), (021), (110), (211), (201), (310), (201), (111), (200), (112), (321), (210), (211), (220), (211), and (221). The XRD pattern of the *Balanites aegyptiaca*-based SrO is consistent with that reported in previous studies, like the Godlaveeti et al. study^35^. The diffractograms of SrO-BC illustrated the distinctive XRD peaks of SrO with almost the same crystallinity as the pure one, owing to the good crystallinity of the *Balanites aegyptiaca*-derived BC. Furthermore, the diffraction peaks of BC did not emerge in the SrO-BC pattern because of its lower mass ratio in the composite matrix. The SrO-BM diffractogram clarified a noticeable diminution in the intensity of the XRD peaks of SrO because of the semi-crystalline nature of BM.

#### FTIR

Figure 3B shows the FTIR spectra of BM, BC, SrO, SrO-BC, and SrO-BM. The BM spectrum observed a broad band between 3417 and 3366 cm^− 1^, which belongs to –OH functional groups of H_2_O or other phytoconstituents^36^. The peaks in the wave number regions of 2849 and 2916 cm^− 1^ are related to the asymmetric and symmetric C–H^37^. The strong peak at 1045 cm^− 1^ is likely due to the stretching of C–O bonds associated with oxygenated functional groups of cellulose, hemicellulose, and lignin^38^. FTIR spectrum of biochar revealed the –OH peak at 3422 cm^− 1^, and the C–H stretching peak appeared at 2923 cm^− 1^. In addition, the corresponding peak to C = C manifested at a wavenumber of 1618 cm^− 1 39^. The SrO spectrum denoted transmittance peaks at 583 and 705 cm^− 1^, corresponding to the Sr-O bond, and the Sr-O-Sr bond appeared at 1032 cm^− 1 40^. SrO-BM and SrO-BC implied the integration of SrO with BM and BC, respectively, confirming the successful manufacturing of the green composites-based *Balanites aegyptiaca.*

#### Surface charge

The zeta potential measurements for SrO-BC and SrO-BM composites at different pH media ranging from 4 to 11 are illustrated in Fig. 3C, D. The ZP curves of SrO-BC and SrO-BM demonstrated that their zero-charge points are 4.11 and 4.16 eV, respectively. These findings indicated the presence of active cationic surfaces of SrO-BC and SrO-BM in highly acidic media. Contrariwise, the surfaces of the composites have anionic active sites at weak acidic and alkaline media.

#### XPS

**Fig. 4:**
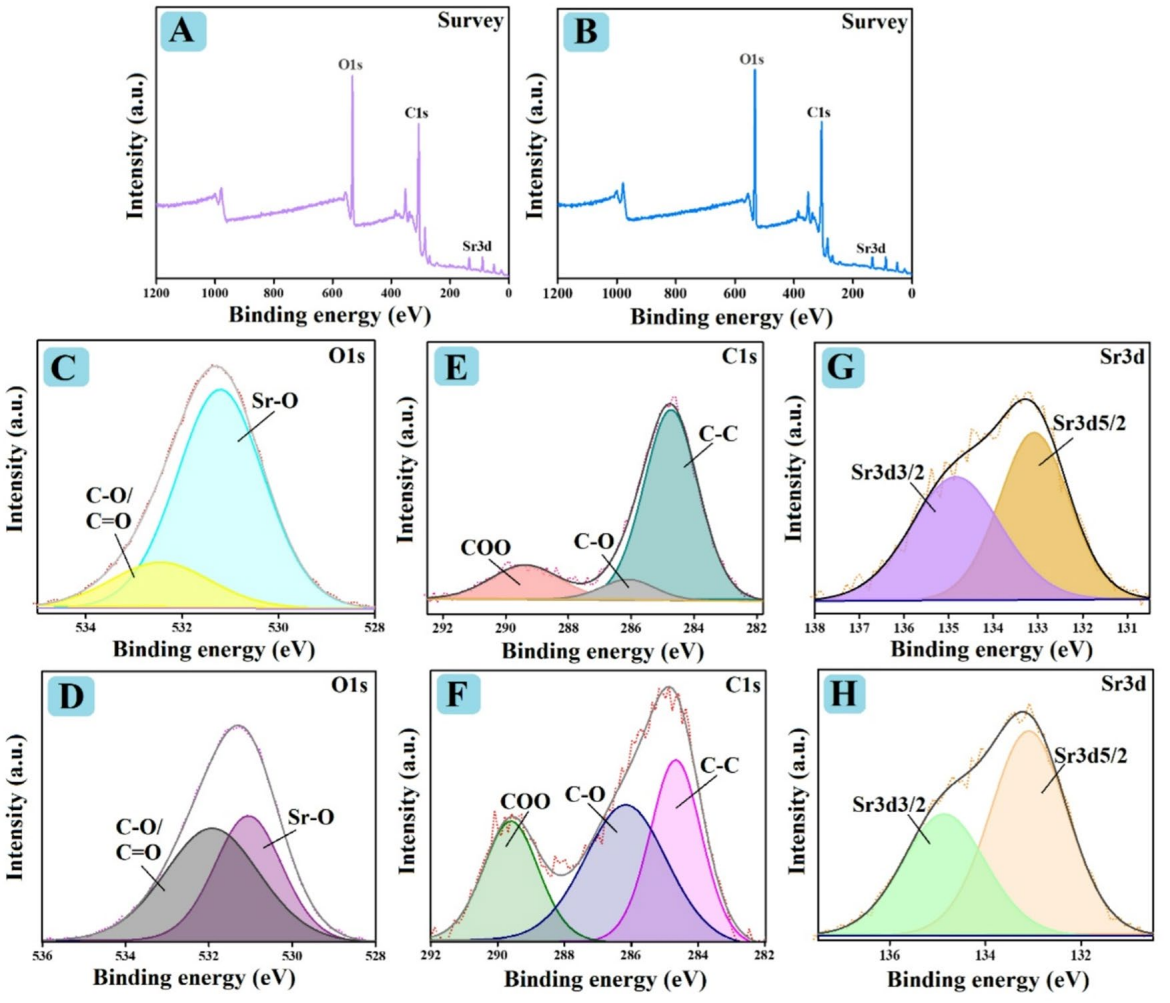
XPS of spectra SrO-BC (**A**) survey, (**C**) O1s, (**E**) C1s, and (**G**) Sr3d and SrO-BM (**B**) survey, (**D**) O1s, (**F**) C1s, and (**H**) Sr3d.

The elemental compositions of SrO-BM and SrO-BC are identified by XPS, as demonstrated in Fig. 4. The survey spectra of SrO-BC and SrO-BM clarified that both composites consist of carbon, oxygen, and strontium (Fig. 4A, B). The O1s spectra (Fig. 4C, D) elucidated the peaks of Sr-O and C − O/C = O at 531.20 and 532.45 eV in SrO-BC and 531.04 and 51.92 eV in SrO-BM. Furthermore, the C1s spectra of SrO-BC and SrO-BM manifested three XPS peaks of the carbon-containing functional groups of C − C, C − O, and COO at 284.53, 287.95, and 289.45 eV and 284.68, 286.17, and 289.17 eV, as represented in (Fig. 4E, F). The Sr3d spectra (Fig. 4G, H) illustrated the peaks of Sr3d5/2 and Sr3d3/2 at 133.09 and 134.84 eV for SrO-BC and 133.09 and 134.86 eV for SrO-BM.

### Optimization of o-NP adsorption

#### Comparison test

**Fig. 5:**
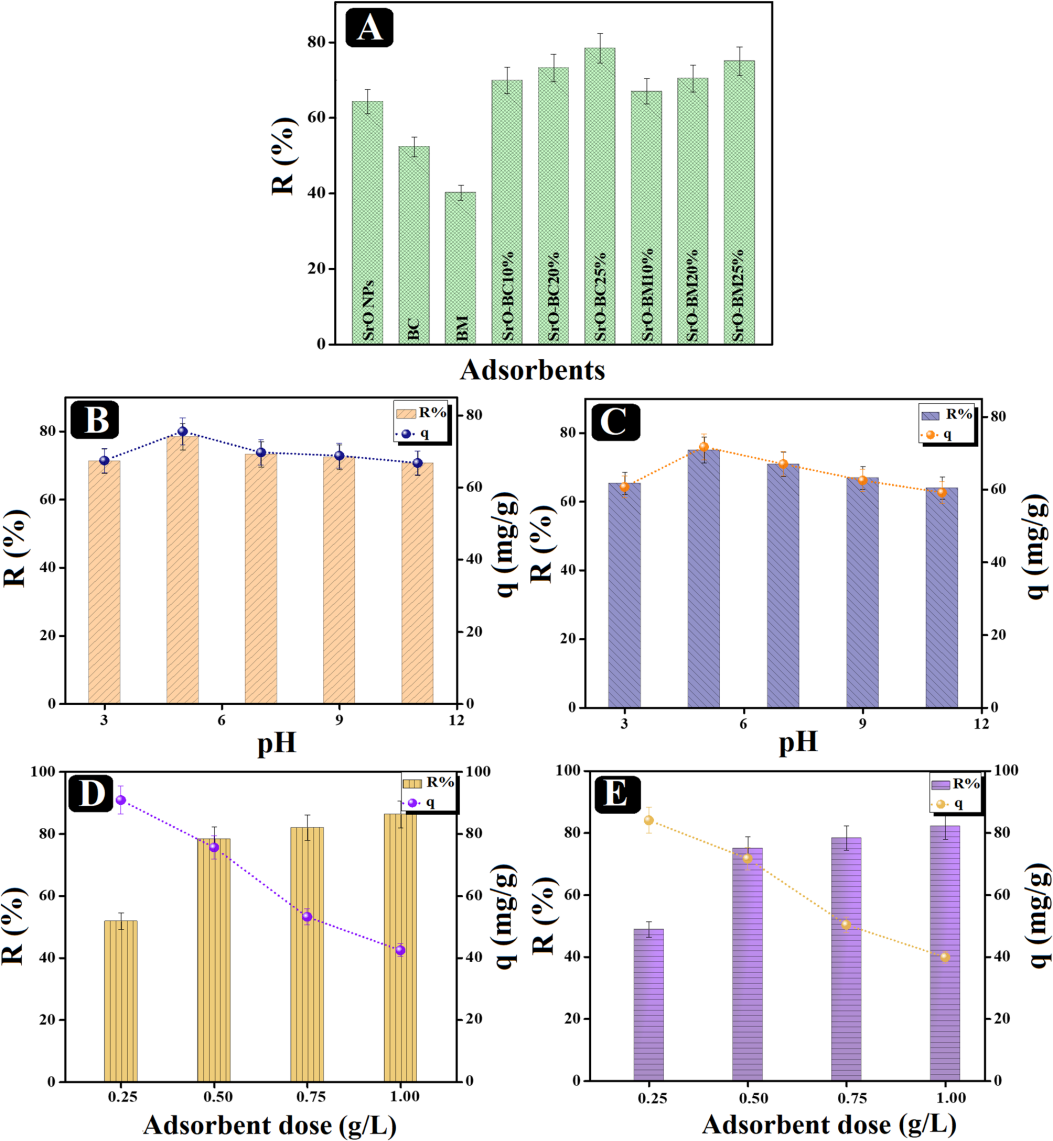
(**A**) Comparison study between the removal aptitude of o-NP by SrO, BC, BM, SrO-BC composites, and SrO-BM composites. (**B**) Effect of pH on adsorbing o-NP by (**C**) SrO-BC and (**D**) SrO-BM. Effect of varying the doses of (**D**) SrO-BC and (**E**) SrO-BM on the removal % of o-NP.

A comparison study was conducted between the removal aptitude of SrO, BC, and BM towards o-NP, clarifying that the adsorption % of o-NP was 64.30, 52.35, and 40.22% by SrO, BC, and BM, sequentially (Fig. 5A). Furthermore, the SrO-BC and SrO-BM composites were post-synthesized with three different percentages of biochar and biomass and then subjected to a comparison test to evaluate the enhancement of the adsorption efficiency of SrO NPS after the addition of biochar or biomass. The removal % of o-NP by the composites is directly proportional to the amount of the loaded biochar or biomass to the surface of SrO NPs as the removal % of 10, 20 and 25% composites were 69.89, 73.23, and 78.42% for SrO-BC and 66.99, 70.44, and 75.04% for SrO-BM, respectively. In light of the experimental results, there was a synergistic effect between SrO NPs with BC and BM separately. In addition, the SrO-BC and SrO-BM composites with 25 wt% of BC and BM, respectively, were chosen for further batch trails.

#### Effect of pH on nitrophenol adsorption

The outcome of the experimental test that involved conducting the o-NP adsorption onto SrO-BC and SrO-BM at different pH media (Fig. 5B, C) implied that the optimum pH of both processes was pH = 5. The findings clarified an amelioration in the R% of o-NP from 71.29 to 78.42% by SrO-BC, while in the case of using SrO-BM, the o-NP adsorption enhanced from 60.67 to 71.68% by raising pH from 3 to 5. In addition, elevating the pH over 5 declined the R% of o-NP by SrO-BC and SrO-BM to 66.75 and 59.10% at pH = 11. Meanwhile, o-NP dwells in its molecular state at pH < 7.23, while beyond pH = 7.23, the o-NP molecules exist in an anionic state^1,42^. Thus, o-NP exists in its molecular form at pH = 5, meaning that electrostatic interaction could not contribute to the o-NP adsorption onto SrO-BC and SrO-BM. Other interactions, such as electron donor-acceptor, π-π interaction, coordination bond, and hydrogen bond, are the dominant forces in these processes^2^. Nonetheless, the diminution in the removal aptitude of o-NP in the alkaline media is explained by the electrostatic repulsion between the anionic o-NP molecules and the anionic adsorption groups on the SrO-BC and SrO-BM surfaces^3^.

#### Effect of dose

Successive o-NP adsorption tests were performed using varied doses of SrO-BC and SrO-BM, as depicted in (Fig. 5D, E). The o-NP removal efficiency by SrO-BC and SrO-BM boosted from 51.88 to 48.91% to 86.34 and 82.17% by increasing the composite dosage between 0.25 and 1.0 g/L, respectively. This increase in the o-NP removal efficiency is most likely due to improving the accessible binding sites by incrementing the mass of SrO-BC and SrO-BM. On the other side, this mass increase resulted in dwindling the uptake capacity of o-NP molecules onto SrO-BC and SrO-BM from 90.79 to 84.04 mg/g to 42.25 and 39.88 mg/g. This observation may be due to the high unoccupied binding groups compared to the concentrations of the o-NP molecules^43^.

#### Optimizing the system temperature

**Fig. 6:**
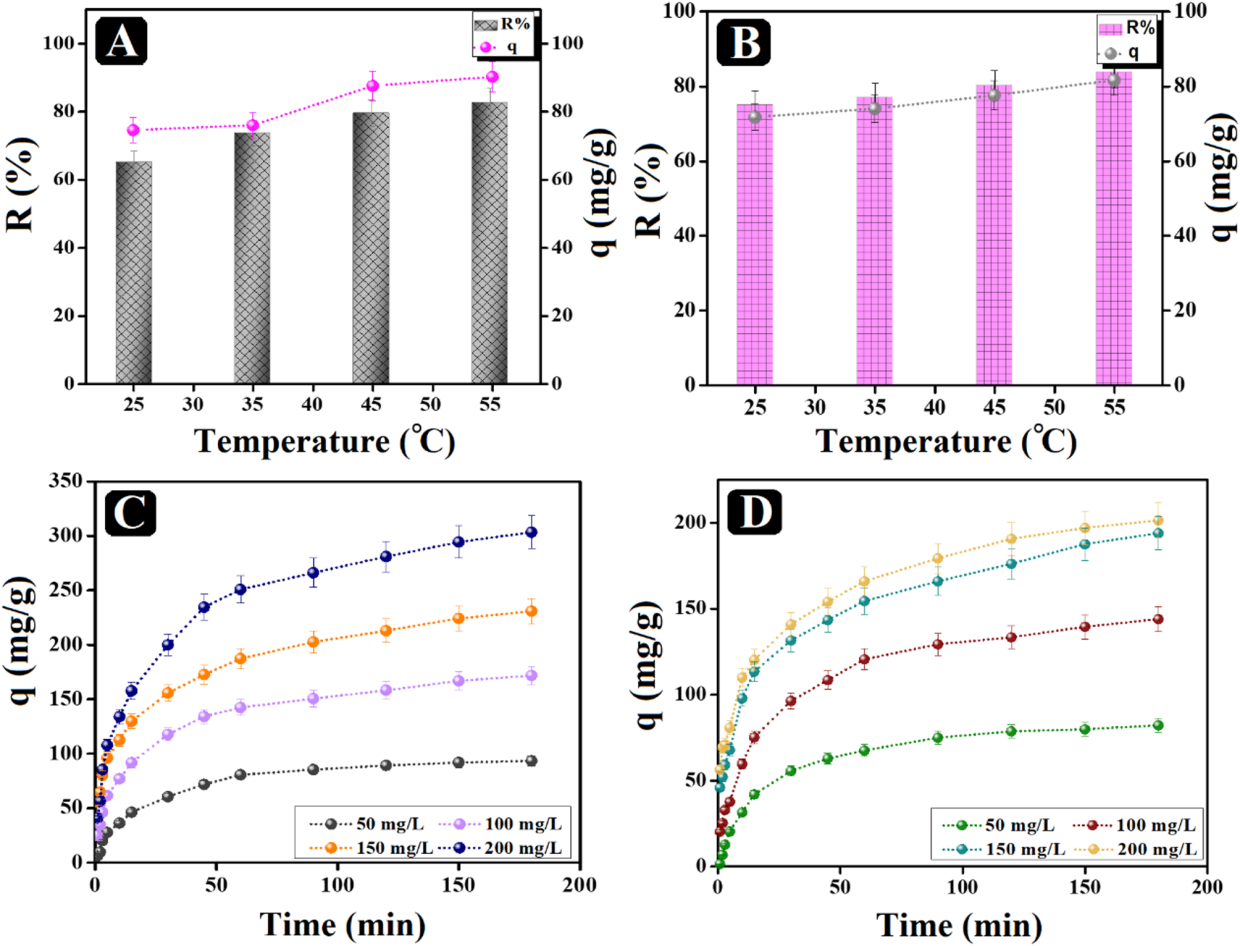
Effect of process temperature on adsorbing o-NP by (**A**) SrO-BC and (**B**) SrO-BM. Effect of elevating the o-NP concentrations on the o-NP adsorption aptitude onto (**C**) SrO-BC and (**D**) SrO-BM.

The adsorption system temperature is another controlling factor in the capacity of the adsorption process, so the adsorption of o-NP onto SrO-BC and SrO-BM was studied at diverse temperatures, as illustrated in (Fig. 6A, B). The adsorption capacity of SrO-BC composite toward o-NP increased from 75.5 to 190 mg/g by raising the temperature from 25 to 55 °C, and that of SrO-BM elevated from 71.68 to 81.57 mg/g. In addition, the removal % of o-NP by SrO-BC improved from 78.41 to 88.02%, while in the case of SrO-BM, it increased from 75.04 to 83.76%. The results could be interpreted by reinforcement of o-NP molecules dispersion, besides the acceleration in their diffusion rates toward the surface of the composites^44^.

#### Optimizing the equilibrium time

The equilibrium adsorption time of the o-NP adsorption processes onto SrO-BC and SrO-BM were examined at a wide scale of o-NP concentrations, as elucidated in Fig. 6C, D. The o-NP adsorption processes onto SrO-BC and SrO-BM fulfilled state of equilibrium after 120 min with a maximal experimental adsorption capacity of 303.37 and 201.35 mg/g, respectively. Notably, an amelioration in the capacity of the adsorbed o-NP molecules onto the SrO-BC and SrO-BM surfaces by elevating the o-NP concentrations from 50 to 200 mg/L. This expected finding may be due to forcing the o-NP deriving forces in the adsorption system by elevating its concentration; thus, these forces could surpass the transfer resistance of the o-NP molecules towards the composites^41^ .

### Isotherm study

**Fig. 7:**
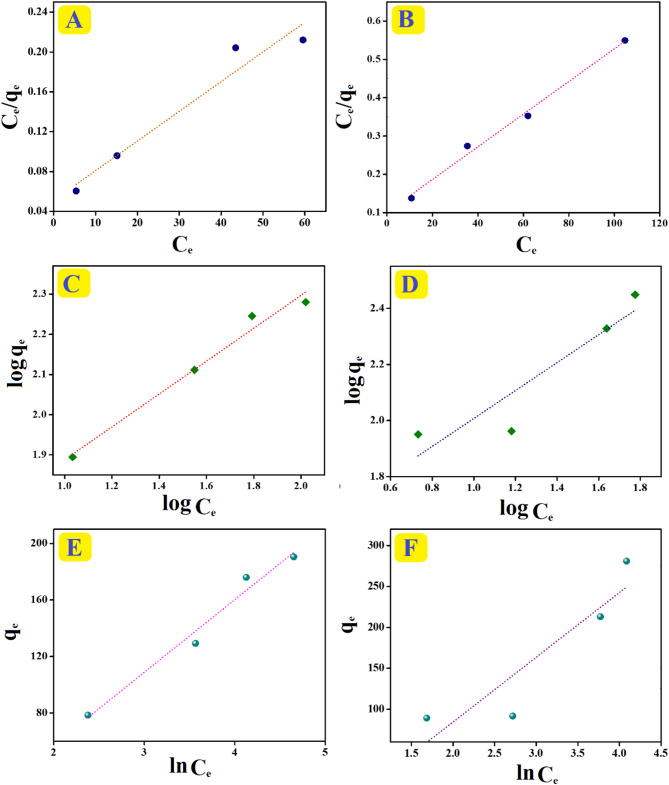
Langmuir, Freundlich, and Temkin curves of the o-NP adsorption processes: (**A**, **C**, **E**) onto SrO-BC and (**B**, **D**, **F**) onto SrO-BM.

The study of isotherms provides information regarding the interaction forces between o-NP and the SrO-BC and SrO-BM composites separately. Langmuir, Freundlich, and Temkin isotherm equations (Table S1) were applied to investigate the dominant interaction force between o-NP and the composites is physical or chemical force^45^. Table 1 lists the resultant parameters acquired from the isotherm curves (Fig. 7A–F). It is obvious from the data that the o-NP adsorption process onto the SrO-BC composite fits the Freundlich model with a correlation value of 0.979, indicating the multilayer o-NP adsorption on the heterogeneous SrO-BC surface^46^. The computed maximum capacity of the adsorbed o-NP onto SrO-BC by Langmuir was 232.74 mg/g. On the other side, the adsorption of o-NP onto SrO-BM is more likely to obey the Langmuir model with a correlation value of 0.996, which suggests governing the chemical adsorption forces on the o-NP adsorption onto SrO-BM with a maximal capacity reached 335.57 mg/g. The b values of the Temkin model were less than 80 kJ/mol, denoting the domination of the physical adsorption forces on the o-NP adsorption onto SrO-BC and SrO-BM^47^. The SrO-BC and SrO-BM composites are suitable adsorbents to adsorb the o-NP onto their surfaces, where the n values of Freundlich are higher than unity.

**Table 1:** The isotherm parameters of the o-NP adsorption processes onto the SrO-BC and SrO-BM composites.

Models	Langmuir			Freundlich			Temkin		
Parameters	q_m_ (mg/g)	b (L/mg)	R^2^	k_F_ ((mg/g)(L/mg)^1/n^)	n	R^2^	A (L/g)	b (kJ/mol)	R^2^
SrO-BC	335.57	0.058	0.926	30.16	2.45	0.971	0.411	0.048	0.966
SrO-BM	234.74	0.042	0.988	32.38	2.01	0.789	0.342	0.031	0.750

### Kinetic study

**Fig. 8:**
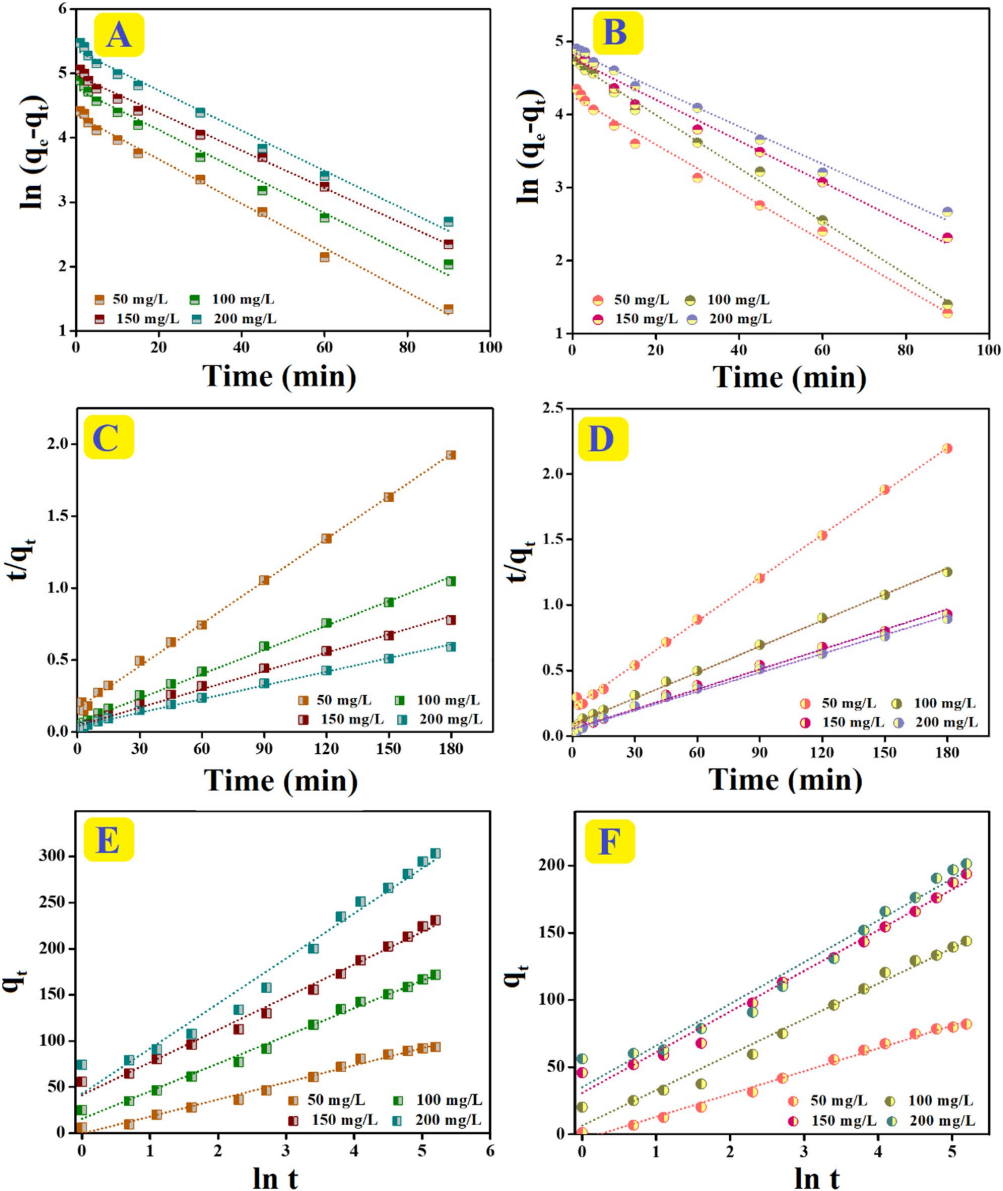
Pseudo-first-order, pseudo-second-order, and elovich curves of the o-NP adsorption processes: (**A**, **C**, **E**) onto SrO-BC and (**B**, **D**, **F**) onto SrO-BM.

The Pseudo-First-Order, Elovich, and Pseudo-Second-Order equations (Table S2) were applied to inspect the experimental adsorption data of o-NP adsorption onto SrO-BC and SrO-BM. The parameters derived from these kinetic studies are summarized in Table 2. From the correlation coefficient (R^2^) of the kinetic curves (Fig. 8A–F), the o-NP adsorption processes onto SrO-BC and SrO-BM obeyed the Pseudo-Second-Order model since the R^2^ values are analogously greater than those of Pseudo-First-Order. Furthermore, the resultant calculated q_e_ of o-NP from Pseudo-Second-Order applied to SrO-BC and SrO-BM are close to the experimental q_e_ compared with those of Pseudo-First-Order. Moreover, the Elovich model manifested the favourability of the adsorption processes of o-NP, as the rate of the o-NP molecules during the adsorption onto SrO-BC or SrO-BM is faster than their desorption rate^48^.

**Table 2:** The kinetic parameters of the o-NP adsorption processes onto the SrO-BC and SrO-BM composites.

Adsorbents	C_o_ (mg/L)	Pseudo-first-order				Pseudo-second-order			Elovich		
		q_e, exp_	q_e, cal_	k_1_	R^2^	q_e, cal_	k_2_	R^2^	α	β	R^2^
SrO-BC	50	89.21	77.47	0.034	0.992	101.73	5.9 × 10^− 4^	0.998	18.12	0.054	0.987
	100	158.20	116.74	0.032	0.985	177.94	4.8 × 10^− 4^	0.996	50.74	0.033	0.993
	150	213.03	143.88	0.029	0.992	235.85	4.2 × 10^− 4^	0.993	114.01	0.028	0.988
	200	280.89	212.51	0.031	0.987	315.46	2.6 × 10^− 4^	0.996	117.83	0.020	0.972
SrO-BM	50	78.43	69.89	0.033	0.989	90.75	5.6 × 10^− 4^	0.999	13.46	0.059	0.992
	100	133.26	112.51	0.037	0.995	151.29	4.9 × 10^− 4^	0.997	33.34	0.038	0.981
	150	175.96	117.49	0.028	0.979	197.24	4.8 × 10^− 4^	0.993	83.80	0.033	0.985
	200	190.56	129.93	0.026	0.989	207.90	4.4 × 10^− 4^	0.993	95.91	0.032	0.967

### Thermodynamic study

**Fig. 9:**
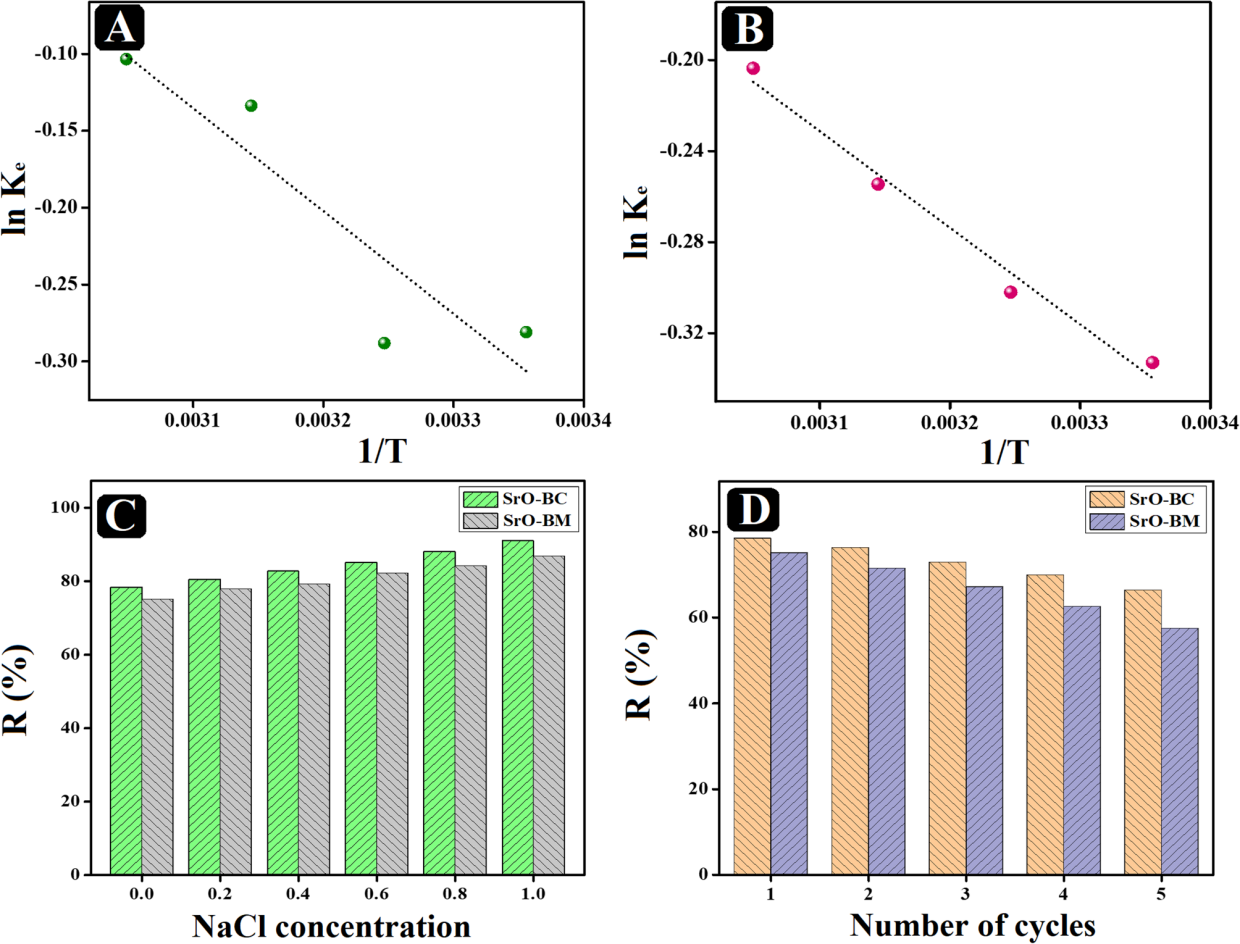
Van’t Hoff curves of the o-NP adsorption onto (**A**) SrO-BC and (**B**) SrO-BM. (**C**) The impact of ionic strength on adsorbing o-NP onto SrO-BC and SrO-BM. (**D**) The cycling test of SrO-BC and SrO-BM during five o-NP adsorption cycles.

The thermodynamic analysis is essential for identifying whether the adsorption manner inside the o-NP/SrO-BC and o-NP/SrO-BM systems are endothermic or exothermic. Therefore, the main thermodynamics parameters were reckoned from Van’t Hoff curves, as illustrated in (Fig. 9A, B). The equations applied on the adsorption data at varied temperatures to determine the change in Gibbs free energy (∆G°), change in entropy (∆S°), and change in enthalpy (∆H°) are mentioned in Text S3. The positive ∆H° magnitudes of the o-NP adsorption onto SrO-BC and SrO-BM indicated controlling the endothermic manner inside both adsorption systems (Table 3). In addition, the ∆H° values denoted dominating the physical interaction on removing the o-NP molecules onto SrO-BC and SrO-BM since ΔH° <80 kJ/mol. These findings were consistent with the ΔG° magnitude that assured the physical adsorption of o-NP onto SrO-BC and SrO-BM, where 0 < ΔG°< – 20 J/mol. Furthermore, the negative values of ΔG° implied the spontaneous adsorption of o-NP onto SrO-BC and SrO-BM. Besides, the ΔS° value of both o-NP adsorption processes was positive, suggesting that the adsorption of nitrophenol on both nanocomposites is an irreversible process, which is conducive to the stability of adsorption^49^.

that adsorption is accompanied by an increase in disorder, with a comparison between adsorption on SrO-BC and SrO-BM^50^.

**Table 3:** Thermodynamic parameters of the o-NP adsorption processes onto SrO-BC and SrO-BM composites.

Adsorbent	Temperature (K)	ΔG° (kJ/mol)	ΔH° (kJ/mol)	ΔS° (J/mol K)
SrO-BC	298	– 4.79	5.56	16.09
	303	– 4.87		
	308	– 4.95		
	313	– 5.03		
SrO-BM	298	– 2.67	3.52	8.99
	303	– 2.72		
	308	– 2.77		
	313	– 2.81		

### Impact of ionic strength

Investigating the effect of ionic strength on the o-NP adsorption aptitude was determined in the presence of NaCl as an electrolyte in the adsorption systems, as shown in Fig. 9C. Surprisingly, the removal efficacy of the o-NP molecules by SrO-BC and SrO-BM enhanced from 78.42 to 71.68% to 91.09 and 84.94%, respectively, by escalating the NaCl concentrations from 0.2 to 1.0 mol/L. This observation is because of the salting-out effect phenomenon, meaning the decline of the o-NP solubility, when o-NP becomes less soluble in the solution it is more likely to bind to the adsorbent material. which spontaneously ameliorates the o-NP removal efficiency^51^.

### Reusability study

To validate the durability of SrO-BC and SrO-BM composites, a reusability study was conducted for five o-NP adsorption runs, as shown in Fig. 9D. The adsorption efficiency of o-NP on the surface of SrO-BC nanocomposite after five consecutive cycles, alongside the performance of the SrO-BM nanocomposite for comparative purposes. It is evident that the removal percentage of both samples decreased following five repeated cycles. This reduction in activity is ascribed to the loss of adsorbent during the collection and rinsing processes in each cycle. Nonetheless, the removal efficiency of o-NP by the SrO-BC nanocomposite remained above 70%, while that of the SrO-BM was above 60% even after five recycling instances, indicating that this innovative nanostructured composite exhibits considerable adsorption stability. In comparison to the SrO-BC sample, the removal percentage of the SrO-BM nanocomposite is lower. This observation may be attributed to the pyrolysis effect on the surface morphology of BM as it transforms into BC, which enhances the adsorption efficiency of the SrO-BC nanocomposite.

### Mechanistic study

**Fig. 10:**
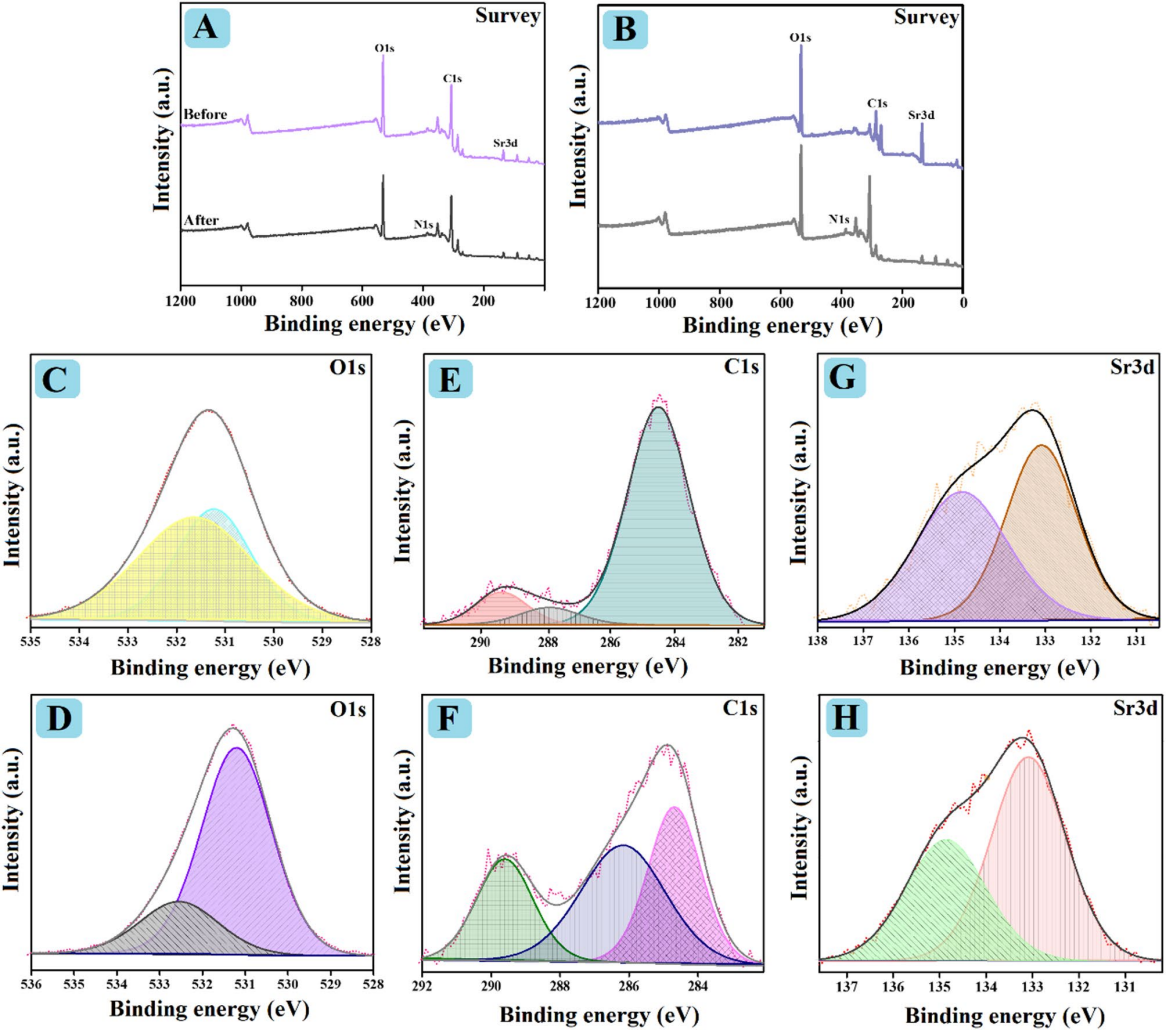
XPS of spectra the used SrO-BC (**A**) survey, (**C**) O1s, (**E**) C1s, and (**G**) Sr3d and the used SrO-BM (**B**) survey, (**D**) O1s, (**F**) C1s, and (**H**) Sr3d.

The XPS survey spectra of the used SrO-BC and SrO-BM composites showed the belonging peak of N1s of o-NP, inferring the occurrence of the o-NP adsorption process, as depicted in Fig. 10A, B. Considering the zeta potential and experimental findings, electrostatic interaction is not involved in the adsorbing mechanism of o-NP onto SrO-BC and SrO-BM. This suggestion owes to the existence of o-NP in its molecular form at pH < 7.23, which transfers to anionic molecules by raising pH over 7.23. Meanwhile, zeta potential reflected that both SrO-BC and SrO-BM carry negative charges at a wide pH scale ranging from 3 to 11. Thereby, electrostatic interaction cannot participate in adsorbing o-NP onto SrO-BC and SrO-BM. Contrariwise, electrostatic repulsion plays an opposite role in hindering the adsorption of the anionic o-NP onto the negatively charged SrO-BC and SrO-BM in the alkaline media.

As confirmed by earlier studied, BM and BC derived from agriculture waste contains mainly cellulose, hemicellulose and lignin which have many hydroxyl groups and benzene rings^52^. As a result, electron donner-acceptor mechanism is a possible adsorption pathway of o-NP, where the electron-rich groups of SrO-BC and SrO-BM, like carboxyl, benzene ring, and hydroxyl can donate electrons to the nitro group of o-NP since it is an electron-acceptor group. The O1s spectra (Fig. 10C, D) clarified shifting in the positions of the oxygen-functional groups’ peaks of SrO-BC and SrO-BM, which indicates their inclusion in the o-NP adsorption process. Hydrogen bonding can enable SrO-BC and SrO-BM to attack o-NP on their surfaces as the hydrogen atoms of the composites can bond to the oxygen and nitrogen atoms of o-NP by hydrogen bonding. Also, the hydrogen atoms of o-NP and oxygen species of SrO-BC and SrO-BM may connected via hydrogen bonds.

**Fig. 11:**
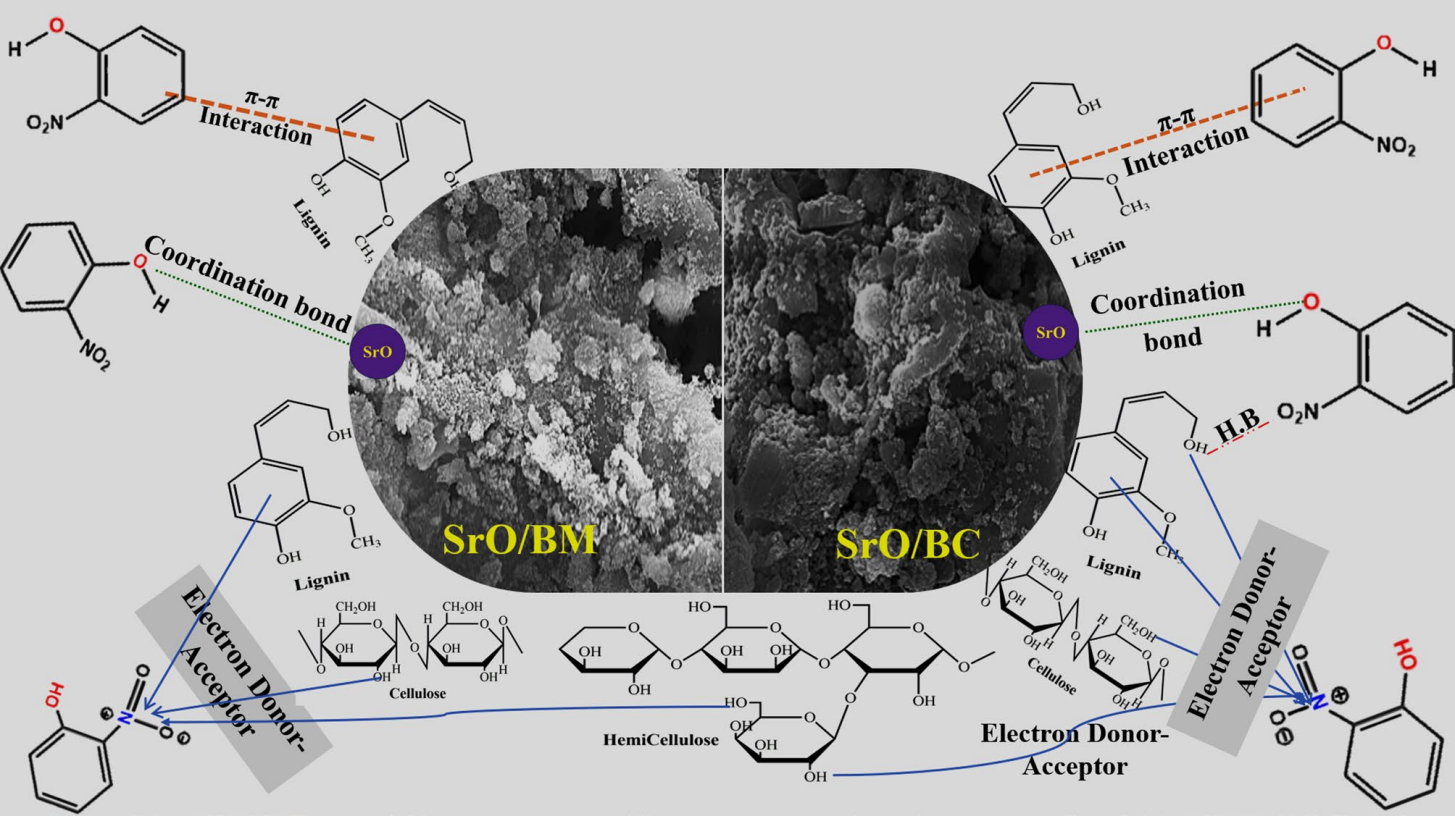
The mechanism of o-NP removal by Sr-BM and Sr-BC composites.

Moreover, the benzene rings of o-NP and both composites can contribute to the adsorption process via the π-π interaction mechanism. The peaks shifting of the C1s spectra confirmed the participation of π-π interaction to adsorb the o-NP molecules onto SrO-BC and SrO-BM, as elucidated in (Fig. 10E, F). Similarly, the peak positions of Sr3d spectra of both SrO-BC and SrO-BM changed after adsorbing o-NP, as depicted in (Fig. 10G, H). This observation is most likely due to the possibility of the coordination bond formation between the Sr species of SrO-BC and SrO-BM and the hydroxyl group of o-NP. Figure 11 illustrates the mechanisms involved in the removal of o-NP by Sr-BM and Sr-BC composites.

**Fig. 12:**
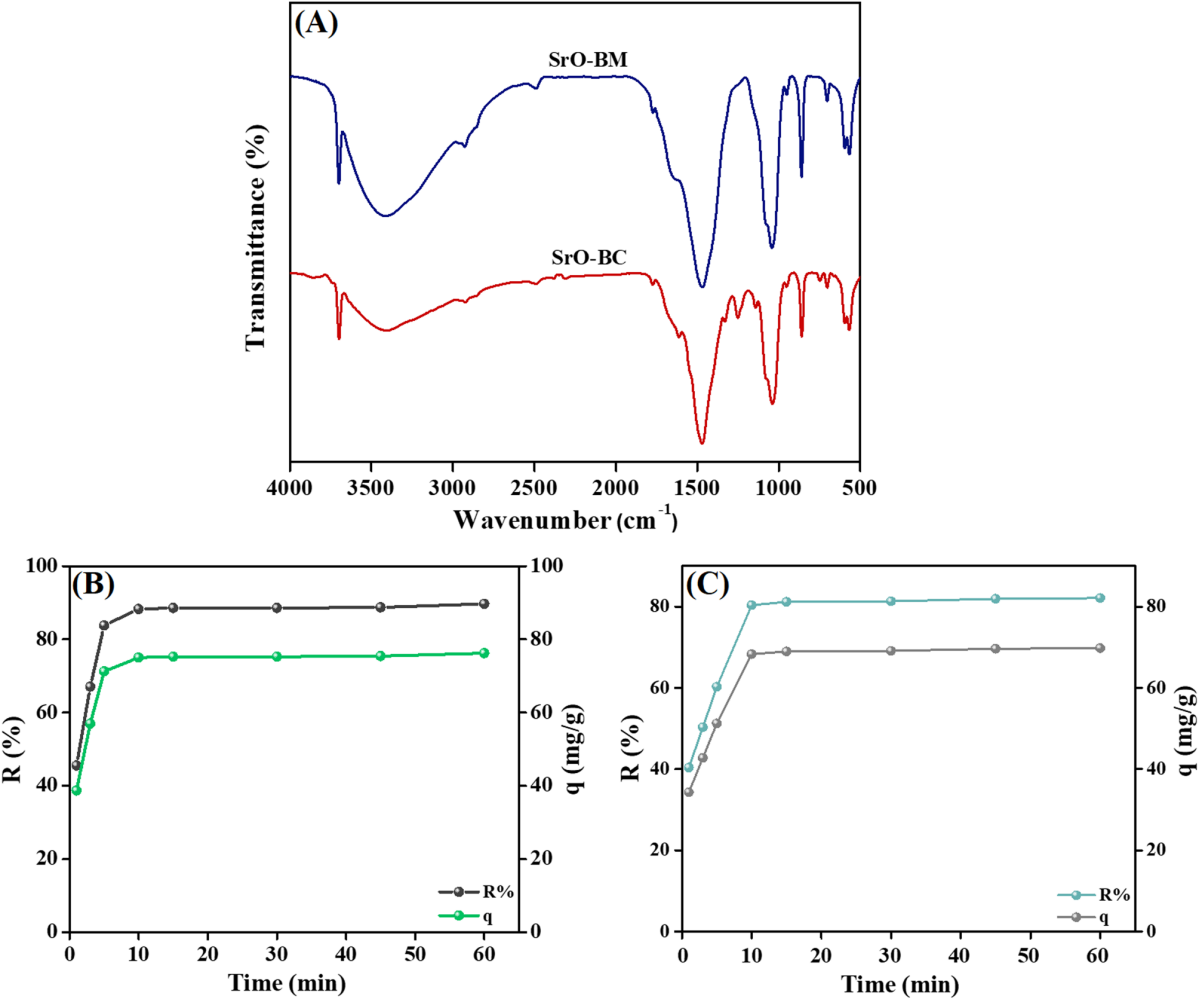
(**A**) FTIR spectra of the used SrO-BC and SrO-BM and the adsorption performance of (**B**) SrO-BC and (**C**) SrO-BM in removing o-NP from real wastewater.

By comparing the FTIR results for both SrO-BC and SrO-bio nanocomposites before and after the adsorption Fig. 12A, it was noticed a little shift in peaks position and a slight change in peaks intensity after the o-NP adsorption process. This observation confirmed occurring the adsorption of the o-NP molecules onto SrO-BC and SrO-BM. Moreover, the FTIR spectra after the adsorption emerged a new peak at 1612.5 cm^[– [1^ in both of SrO-BC and SrO-BM nanocomposite which is attributed to the –N = O stretching.

### Real wastewater test

The test aimed to evaluate the adsorption performance of SrO-BC and SrO-BM in the removing (o-NP) from genuine wastewater. For this purpose, 0.5 g/L of each composite was added to a 20.0 mL sample of real wastewater, which was collected from the effluent of a paint industry in Alexandria, Egypt. The characteristics of the wastewater prior to and following the adsorption process are detailed in Table 4. A comparison of the two composites revealed that SrO-BC exhibited a higher adsorption capacity of 76.2 mg/g, compared to 69.7 mg/g for SrO-BM, when the initial concentration of o-NP was established at 42.5 mg/L, as derived from the calibration curve. Furthermore, the percentage of o-NP removal was found to be 88.5% for SrO-BC and 81.9% for SrO-BM, as depicted in Fig. 12B, C. These findings confirm the efficacy of both SrO-BC and SrO-BM in the elimination of o-NP, with SrO-BC demonstrating a superior adsorption capacity for o-NP in comparison to SrO-BM.

**Table 4:** Wastewater characteristics before and after adsorption.

Parameters	Average values (before treatment)	Average values (after treatment)	
		SrO-BC	SrO-BM
pH	9.68	9.06	8.80
Temperature (ºC)	25	25	25
TDS (ppm)	520	442	495
Turbidity (NTU)	350	299	309
Conductivity (µS/cm)	1040	963	995

### Comparison study

The adsorption capabilities of the SrO-BC and SrO-BM composites toward adsorbing o-NP were compared with the reported adsorbents in previous investigations, as listed in Table 5. The green SrO-BC and SrO-BM composites have revealed higher adsorption capacity than the reported green adsorbents. In addition, the composites have exhibited a competitive adsorption performance compared to the expensive chemical-based adsorbents that may result in pollution during fabrication.

**Table 5:** Comparison between the capabilities of the SrO-BC and SrO-BM composites toward adsorbing o-NP compared with other adsorbents.

Adsorbents	q_max_ (mg/g)	Refs.
Hizikia fusiformis-derived BC	10.39	^4^
Fe_3_O_4_/AP-coke/N-Cs	291.55	^5^
Cr-BDC	310.00	^6^
Date pits-derived AC	142.9	^7^
MoO_3_/ZIF-67/AmGO	500.00	^8^
WRPC-1.25	393.42	^14^
Hyacinth-derived AC	47.62	^53^
GO/COFs	438.31	^54^
Fe_3_O_4_-κ-Carr/MIL-125(Ti)	320.26	^2^
Opuntia ficus-indica	25.60	^55^
SrO-BC SrO-BM	335.57 234.74	This study

## Cost analysis

After deducing the high adsorbability and recyclability of SrO-BC and SrO-BM, it was essential to conduct a cost analysis, as clarified in Table 6. The cost analysis denoted the cost-effectiveness of SrO-BC and SrO-BM, suggesting their application on an industrial scale.

**Table 6:** The cost analysis of the SrO-BC and SrO-BM composites.

Category	Details	Cost (USD)
Raw materials	*Balanites aegyptiaca*	−
	SrCl_2_.6H_2_O (70.6 $/500 g)	2.66 g = 0.38 $
	NaOH (6.64 $/500 g)	2.00 g = 0.027 $
	C_2_H_5_OH (87.40 $/1 L)	100 mL = 8.74 $
Processing	Electricity for muffle furnace, oven, magnetic stirrer, and balance	5.00 $ for SrO-BM 9.50 $ for SrO-BC
Waste management	Disposal of chemical waste	2.00 $
Additional (QC)	XRD, FTIR, SEM, XPS, and Zeta Potential	59.00 $ for SrO-BM 59.00 $ for SrO-BC
Total (lab scale)		SrO-BM = 75.15 $/batch SrO-BC = 79.65 $/batch

## Conclusion

The sustainable SrO-BC and SrO-BM composites were synthesized successfully from collected *Balanites aegyptiaca* from Sudan. The XRD analysis confirmed the crystalline characteristics of the SrO-BC and SrO-BM composites. The zeta potential curves of SrO-BC and SrO-BM clarified that their zero-charge points are 4.11 and 4.16 eV, respectively. The experimental work suggested the ineffective role of electrostatic interactions in the o-NP adsorption onto SrO-BC and SrO-BM. In contrast, the electrostatic repulsion oppositely impacted the adsorption aptitude of o-NP in the basic media. The computed maximal capacities of the adsorbed o-NP onto SrO-BC and SrO-BM by Langmuir are 335.57 and 234.74 mg/g, respectively, at pH = 5 and processing temperature = 25 °C. The isotherm assessments elucidated the suitability of Freundlich and Langmuir to model adsorbing o-NP onto SrO-BC and SrO-BM, respectively. The kinetic assessments clarified the preference of Pseudo-Second-Order to represent the o-NP adsorption onto SrO-BC and SrO-BM. Several pathways were supposed to control the adsorption mechanisms of the o-NP molecules onto SrO-BC and SrO-BM, including electron donner-acceptor, hydrogen bond, pi-pi interaction, and coordination bond. Finally, SrO-BC exhibited the best adsorption performance toward o-NP and recycling ability compared with SrO-BM, which clarified the significance of converting BM to BC.

## Supplementary Information

Below is the link to the electronic supplementary material.

Supplementary Material 1

## Data Availability

The datasets used and/or analysed during the current study available from the corresponding author on reasonable request.
