# Supplementary material for: Engineering of balanites aegyptiaca-derived SrO@biochar and SrO@biomass for nitrophenol removal from wastewater
Source: Sci Rep. 2025 Dec 15;15:43869. doi: 10.1038/s41598-025-26771-x (PMC12708860; doi:10.1038/s41598-025-26771-x)
Supplement: Supplementary file 1 — Supplementary Material 1 [file 41598_2025_26771_MOESM1_ESM.docx]

**Engineering of Balanites aegyptiaca-derived SrO@Biochar and SrO@Biomass for Nitrophenol Removal from Wastewater**

Manal Fawzy^1,2,*^, Rania Alzain^1^, Abdelazeem S. Eltaweil^3,4,*^,Eman M. Abd El‑Monaem^5^

^1^Environmental Sciences Department, Faculty of Science, Alexandria University, 21511, Alexandria, Egypt.

^2^Green Technology Group, Faculty of Science, Alexandria University, 21511, Alexandria, Egypt.

^3^Department of Engineering, Faculty of Technology and Engineering, University of Technology Applied Sciences, Sultanate of Oman.

^4^ Department of Chemistry, Faculty of Science, Alexandria University, Alexandria 21321, Egypt

^5^Advanced Technology Innovation, Borg El-Arab, Alexandria, Egypt

* Corresponding author: Manal Fawzy ([dm_fawzy@yahoo.com](mailto:dm_fawzy@yahoo.com))

Abdelazeem Eltaweil ([abdelazeemeltaweil@alexu.edu.eg](mailto:abdelazeemeltaweil@alexu.edu.ehg))

Authors’ emails: [rania-alzain@hotmail.com](mailto:rania-alzain@hotmail.com)

[emanabdelmonaem5925@yahoo.com](mailto:emanabdelmonaem5925@yahoo.com)

**Text S1.** The used chemicals in fabricating SrO-BC and SrO-BM composites.

Strontium chloride hexahydrate (SrCl_2_.6H_2_O) was purchased from Aladdin Industrial Corporation, China. Sodium Hydroxide (NaOH), ethanol (C_2_H_6_O), and sodium Chloride (NaCl) were provided from Sinopharm Chemical Reagents, China. o-nitrophenol was supplied from Sigma-Aldrich, USA.

**Text S2.** The applied characterization instruments.

The morphology, chemical composition, magnetism and surface charge of SrO, BC, BM, SrO-BC, and SrO-BM were investigated using Fourier Transform Infrared (Frontier, PerkinElmer, FTIR), Scanning Electron Microscope (JEOL 7500F, SEM), X-Ray Photoelectron Spectroscopy (ESCALAB 250XI, XPS), Zeta Potential (Malvern, ZP), and X-Ray Diffraction (PANalytical, XRD),

**Table S1.** Non-linear equations of the applied adsorption isotherm models

| **Model** | **Equation** |
| --- | --- |
| **Langmuir** | $\frac{C_{e}}{q_{e}}=\frac{1}{K_{L} q_{m}}+\frac{C_{e}}{q_{m}}$ **(1)** |
| **Freundlich** | $loglog q_{e} =loglog K_{F} +\frac{1}{n}loglog C_{e}$ **(2)** |
| **Temkin** | $q_{e}=B lnA+Blnln C_{e}$ **(3)** |

Where, q_m_ and K_L_ are the maximum monolayer adsorption capacity and Langmuir constant, respectively. n and K_F_ are Freundlich constants. A is the equilibrium binding constant and $=\frac{RT}{b}$ , b is Temkin constant related to heat of adsorption.

**Table S2.** Non-linear equations of the applied adsorption kinetic models

| **Kinetic Model** | **Equation** |
| --- | --- |
| **Pseudo-First-Order** | $lnln (q_{e} -q_{t})=lnln q_{e}-k_{1}\left( t \right)$**(4)** |
| **Pseudo-Second-Order** | $\frac{t}{q_{t}}=\frac{1}{k_{2}q_{e}^{2}+ \frac{1}{q_{e}}\left( t \right)}$ **(5)** |
| **Elovich** | $q_{t}=\frac{1}{\beta} ln \left( \alpha\beta\right)+ \frac{1}{\beta}$ ln (t)  **(6)** |

Where, q_t_ and q_e_ are amounts of o-NP uptakes at time t and equilibrium, respectively. k_1_ and k_2_ are the rate constants of Pseudo-First-Order and Pseudo-Second-Order, respectively. Furthermore, α and β are Elovich coefficients that represent the initial adsorption rate and the desorption coefficient, respectively, also related to the extent of surface coverage and activation energy for chemisorption.

**Text S3.** The equations of the thermodynamic parameters.

Change in entropy (ΔSº), change in enthalpy (ΔHº) and change in free energy (ΔGº) were calculated from the following equations.

$lnln K_{e} =\frac{\Delta S^{o}}{R}-\frac{\Delta H^{o}}{RT}$ , $K_{e}=\frac{C_{Ae}}{C_{e}}$ **(7, 8)**

${\Delta G}^{o}={\Delta H}^{o}-T{\Delta S}^{o}$ **(9)**

Where, C_e_ and C_Ae_ are the concentration of o-NP in the bulk solution and onto the surface of MoO_3_/ZIF-67/AmGO at equilibrium, respectively. R and T are gas constant and the system temperature, respectively.
